# Jejunal diverticulum and pneumatosis intestinalis presenting as pneumoperitoneum: A case report

**DOI:** 10.1016/j.ijscr.2023.108320

**Published:** 2023-05-13

**Authors:** Milan KC, Suman Dahal, Sangam Shah, Laligen Awale, Prasan B.S. Kansakar

**Affiliations:** Tribhuwan University Teaching Hospital, Institute of Medicine, Kathmandu, Nepal

**Keywords:** Jejunal diverticulum, Pneumatosis intestinalis, Pneumoperitoneum, Case report

## Abstract

**Introduction and importance:**

Jejunal diverticulum is a rare condition that affects less than 0.5 % of population. Pneumatosis is also a rare disorder marked by gas in the intestinal wall's submucosa and subserosa. Both the conditions are rare cause of pneumoperitoneum.

**Presentation of case:**

A case of 64 years female presented with acute abdomen and upon investigation found to have pneumoperitoneum. Exploratory laparotomy was done and intraoperatively there was multiple jejunal diverticula and pneumatosis intestinalis in separate segments of bowel and closure was done without any resection of bowel segments.

**Clinical discussion:**

Small bowel diverticulosis was considered to be an incidental anomaly; however, it is now thought to be acquired. Pneumoperitoneum is a common complication of diverticula perforation. The occurrence of pneumatosis cystoides intestinalis or subserosal dissection of air around the colon or adjacent structures has been linked to pneumoperitoneum. Complications should be managed accordingly however, occurrence of short bowel syndrome should be considered before doing resection anastomosis of involved segment.

**Conclusion:**

Jejunal diverticula and pneumatosis intestinalis both are rare cause of pneumoperitoneum. Combination of both the condition giving rise to pneumoperitoneum is extremely rare. These conditions can give rise to diagnostic dilemma in clinical practice. One should always think these as differentials when patient with pneumoperitoneum are encountered.

## Highlights

• Pneumoperitoneum rarely can be caused by jejunal diverticula and pneumatosis intestinalis.
• Combination of both pneumatosis intestinalis and jejunal diverticular disease is extremely rare.
• Proper clinical evaluation and investigation need to be done as these conditions may cause dilemma in diagnosis.
• Surgical treatment may also depend on intraoperative findings.

## 1 Introduction

Jejunal diverticulum is a rare condition that affects less than 0.5 % of population [1]. Males are more likely to develop the condition, and the incidence rises with age [2]. Small bowel diverticular disease usually presents with non-specific symptoms. Pneumatosis is also a rare disorder marked by gas in the intestinal wall's submucosa and subserosa. Pneumatosis can be caused by a variety of factors, including lung disease, scleroderma, immunosuppression, and a variety of gastrointestinal disorders. It's also possible that the incidence is rising as a result of iatrogenic factors including gastrointestinal instrumentation and prescribed drugs [3]. Both the cases can give rise to pneumoperitoneum but is very rare. Here we report a case of 64 years old female who presented with acute abdomen and underwent laparotomy for suspected hollow viscus perforation but intra-operatively she was found to have pneumatosis intestinalis with jejunal diverticulosis. This case report has been reported in line with the SCARE Criteria [4].

## 2 Presentation of case

A 64 years female from kathmandu presented to our emergency department with chief complaints of pain in abdomen for 3 days and vomiting for 1 day. The pain in abdomen was generalized all over abdomen and was mild to moderate in intensity. She had undergone laparoscopic cholecystectomy one year back, since then she had abdominal distension and altered bowel habit. She had 3 episodes of vomiting one day back which was non-projectile and contained food particles. She had not passed stool and flatus for 2 days. She had chronic obstructive pulmonary disease (COPD) for one year. She was non-smoker and did not consume alcohol. She had no history of diabetes mellitus, hypertension, or pulmonary tuberculosis.

She was conscious, co-operative, well oriented to time, place, and person. On general physical examination she had dehydration, however, she had no pallor, icterus, cyanosis, or clubbing. Her blood pressure was 102/78 mmHg, pulse 80 beats/ min, and respiratory rate 21 breaths/min. On abdomen examination, her abdomen was soft and distended, mild tenderness over generalized abdomen was present however there was no rebound tenderness or guarding, tympanic on percussion and liver dullness was obliterated. However, there was no organomegaly and bowel sound was not present.

**Fig. 1.1:**
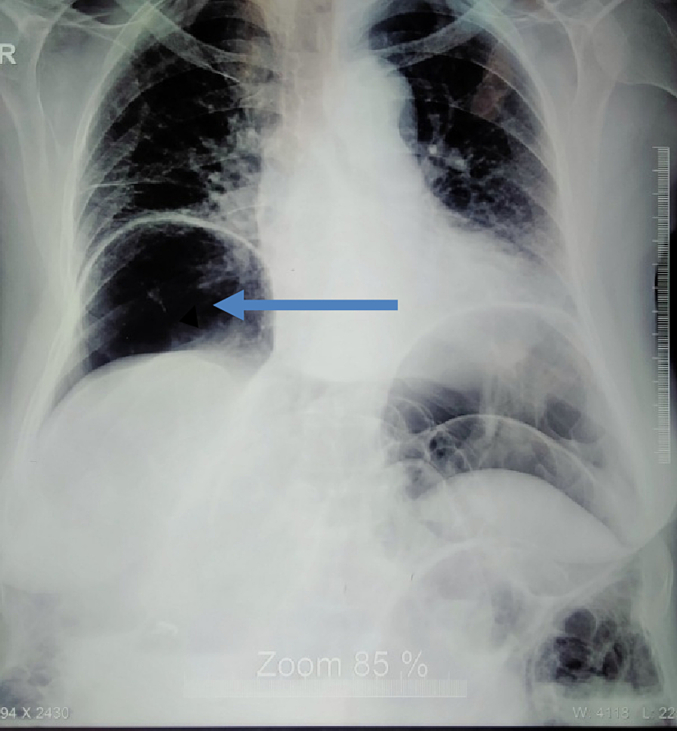
Showed gas under diaphragm.

**Fig. 1.2:**
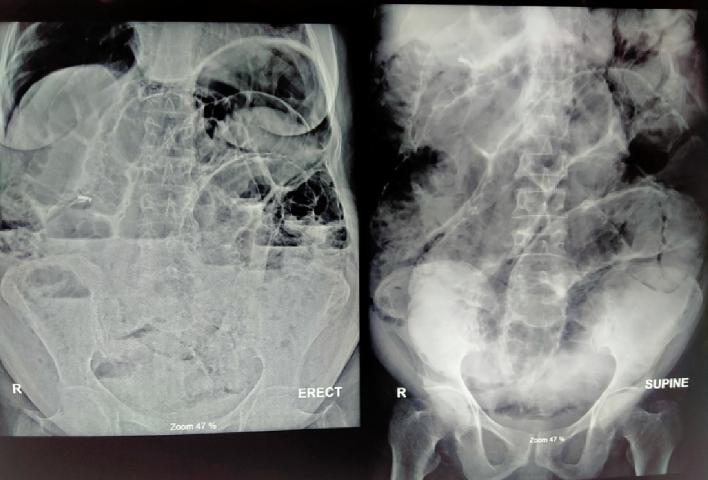
Showed multiple air fluid level in erect and distended bowel in supine abdominal X-ray.

**Fig. 2:**
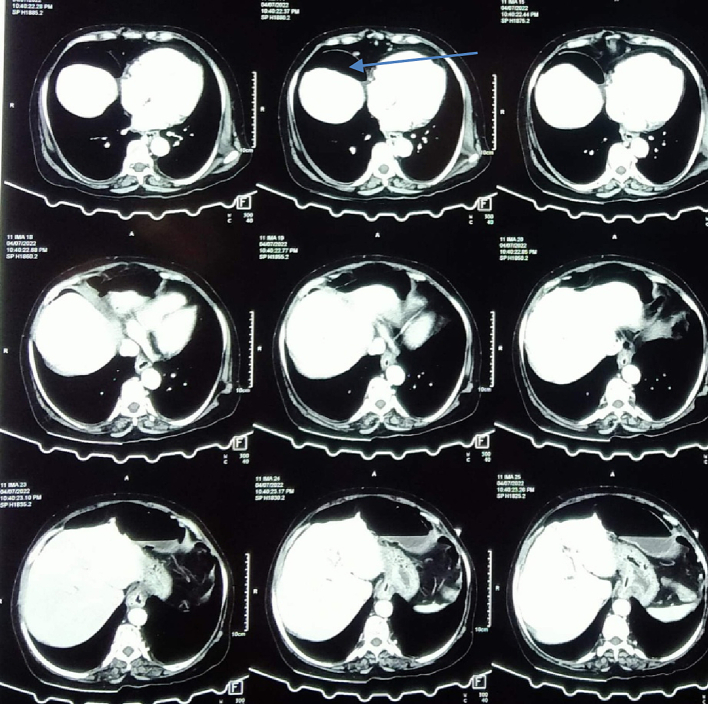
Showed gas under diaphragm.

Her laboratory investigations are shown in Table 1. Ultrasonography (USG) of the abdomen and pelvis showed prominent dilated bowel loops filled with fluid and partially distended urinary bladder. X-ray of chest erect showed Gas under diaphragm over right side (Fig. 1.1) and X-ray abdomen erect and supine showed multiple air-fluid level and distended bowel loops respectively (Fig. 1.2) and CT scan also revealed Gas under diaphragm (Fig. 2).

**Table 1:** Laboratory investigations of the patient.

Lab parameters	Values
Hemoglobin	14.6 g %
Total leucocyte count	2900 cells/mm^3^
Neutrophils	61 %
Lymphocytes	30 %
Platelets	206,000 cells/mm^3^
Random blood sugar (RBS)	106 mg/dl
Sodium	136 mmol/L
Potassium	4 mmol/L
Urea	4.8 mmol/L
Creatinine	93 mmol/L
Total bilirubin	0.8 mg/dl
Direct bilirubin	0.2 mg/dl
Alanine aminotransferase (SGPT)	24 U/L
Aspartate aminotransferase (SGOT)	33 U/L
Alkaline phosphate	90 U/L
Amylase	29 U/L
Prothrombin time (PT)	18 s
International normalized ratio (INR)	1.5
Lactate	1.2

**Fig. 3:**
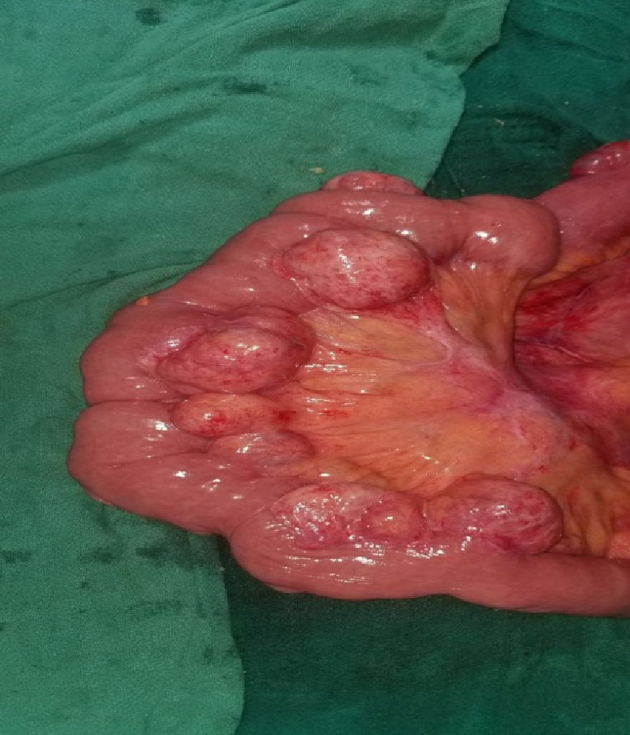
Multiple diverticulum over mesenteric border of jejunum.

**Fig. 4:**
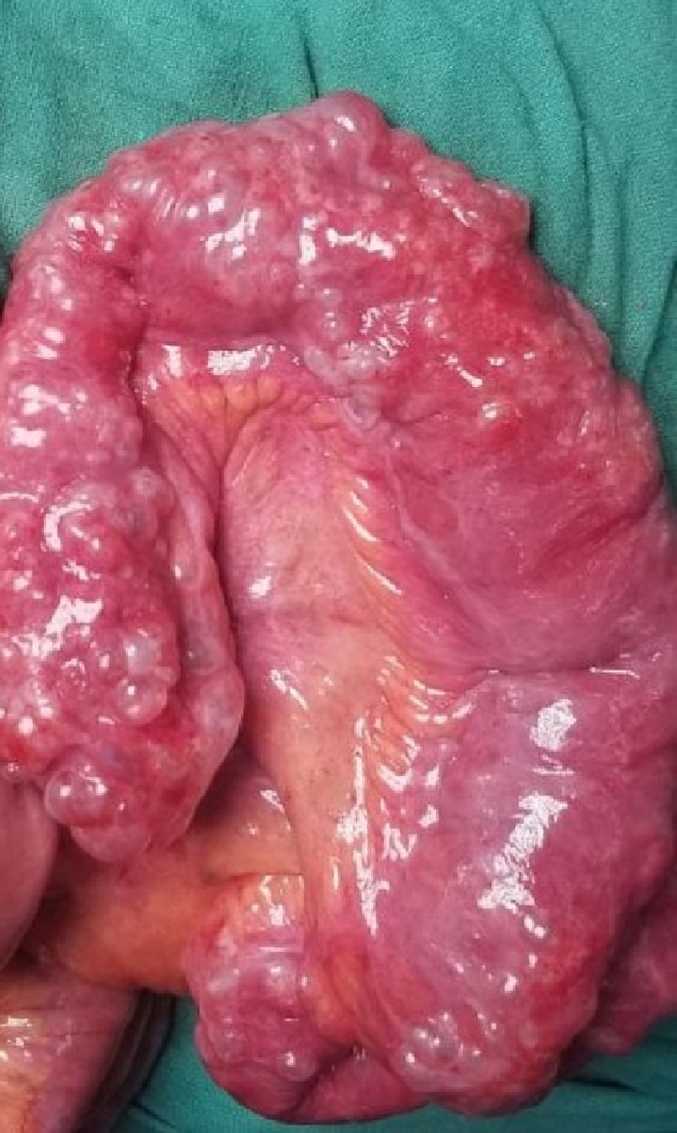
Air filled space in intestinal wall (Pneumatosis intestinalis).

**Fig. 5:**
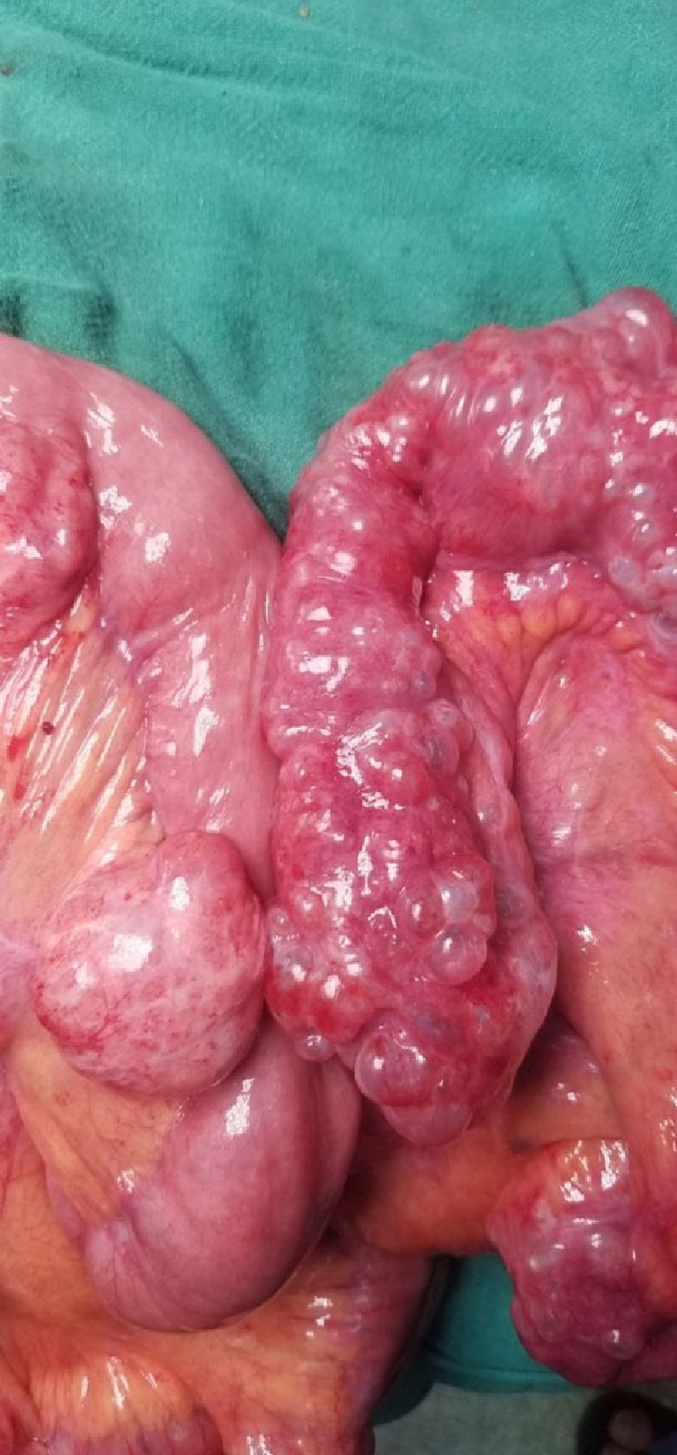
Jejunal diverticula with air filled space in intestinal wall.

Following this she was suspected to have hollow viscus perforation peritonitis with bowel obstruction and was planned for explorative laparotomy. The procedure was performed by above mentioned author team in our tertiary centre in emergency basis. However, intraoperatively there was pneumatosis intestinalis with jejunal diverticulosis (Figs. 3, 4, 5) with no any perforation. Ileocecal junction was normal up to 20 cm, 120 cm proximal to it pneumatosis intestinalis was present, following this 130 cm proximal the bowel was normal then again up to 150 cm proximal jejunal diverticulosis was present. No other visceral perforation was found. Decompression of bowel was done and abdomen was closed. Small specimen of wall of intestine from involved segment was sent for histopathological examination. She was hemodynamically stable after the surgery. On follow-up after 2 weeks there were no issues and the bowel and bladder habit were normal. Patient was satisfied with our treatment and was positive towards our counseling.

**Fig. 6:**
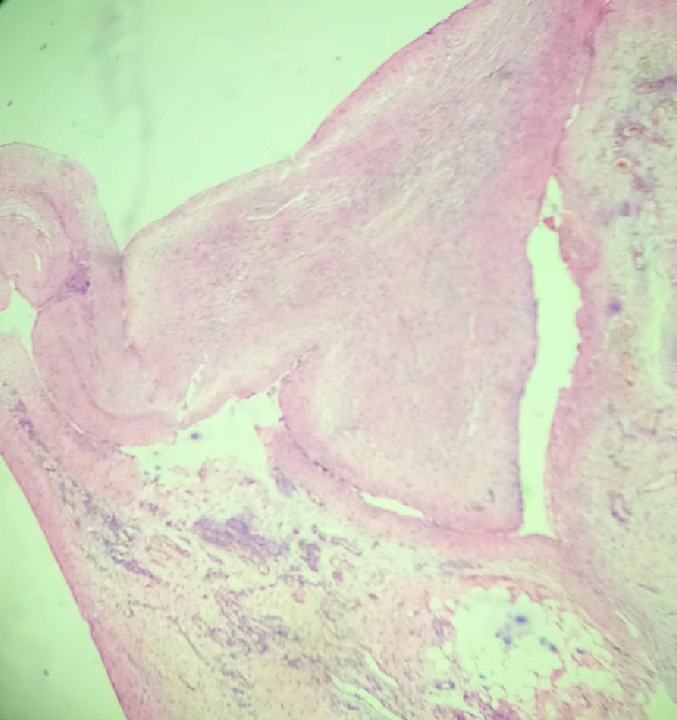
Fibrocollagenous tissue.

**Fig. 7:**
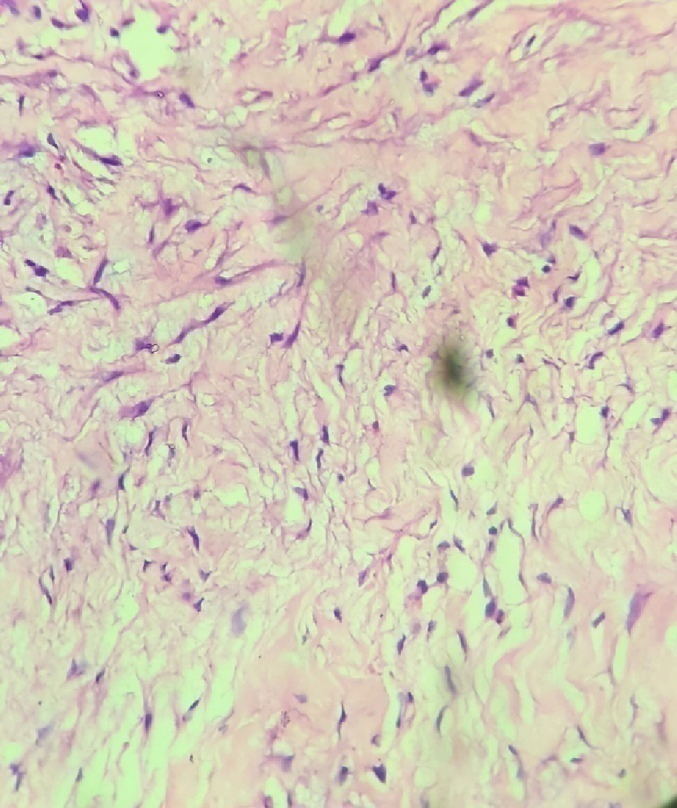
Inflammatory infiltrates.

Histopathological examination showed fibrocollagenous tissue with mild chronic inflammatory infiltrates, vascular proliferation and edematous changes (Figs. 6, 7).

## 3 Discussion

Pneumoperitoneum is a condition characterized by presence of free air in abdominal cavity. Various causes of pneumoperitoneum are perforated peptic ulcers, bowel obstruction, penetrating trauma, ruptured diverticulum, after laparoscopy. Pneumoperitoneum can be diagnosed by plain x ray chest erect showing both dome of diaphragm where free gas is seen below the diaphragm and computed tomography of abdomen and pelvis in doubtful cases. Treatment depends on the causes and all pneumoperitoneum cases do not need surgical treatment because it may be caused by conditions that do not need laparotomy.

Small bowel diverticulosis is false diverticulum defined as thin-walled sacculations made of mucosa, submucosa, and occasionally a thin coating of serosa [5]. These diverticula are mucosal hernias caused by holes in the muscle layers along the paths of the visceral arteries at the site of entry of the vasa recta, which is usually seen on the jejunum's mesenteric border. The pathophysiology of small-bowel diverticula is unknown, however, they are thought to be caused by smooth muscle motor dysfunction or myenteric plexus dysfunction in the small bowel [6]. The size of these diverticula varies from a few millimeters (mm) to more than 10 cm (cm). The high diameter of the penetrating jejunal arteries is linked to the predominance of diverticula in the jejunum [7,8]. Coexisting diverticulosis can be identified in the colon in 20 %–70 % of cases, the duodenum in 10 %–40 % of cases, and the esophagus and stomach in 2 % of cases [5,9]. On autopsy, small intestinal diverticula are found in 0.06 % to 4.6 % [10]. The incidence rate rises with age, reaching a peak in the sixth and seventh decades. Men have been reported to have a larger incidence than women [8], although newer data suggests a possible reversal in the sex distribution [9]. Small bowel diverticulosis was initially considered to be an incidental anomaly; however, it is now thought to be acquired [6,8].

Clinical signs and symptoms include a bloating sensation after eating, as well as vague and chronic epigastric or periumbilical abdomen pain of varying severity. The clinical condition is usually asymptomatic if there is no complication arising from the same condition. Due to the disease's relative clinical rarity and diverse presentation, identification may be delayed or hindered. Diagnosis is frequently determined on the spur of the moment, either through radiographic inspection or laparotomy due to complications.

Chronic stomach pain, malabsorption, bleeding, diverticulitis, blockage, abscesses in the mesentery, and perforation are all reported complications of jejuno-ileal diverticulosis, which affect 10 %–30 % of patients. Diverticulitis with or without perforation is the most prevalent acute complication of the jejuno-ileal diverticula, occurring in 2.3 %–6.4 % of cases [5,7] and with a mortality rate of up to 24 % [11]. This can be caused by gastrointestinal bleeding or bacterial overgrowth, and it can become irritated and inflamed as a result of consumed food, resulting in acute abdominal pain.

Before an exploratory laparotomy or diagnostic laparoscopy, the diagnosis of complex or uncomplicated jejunal diverticulosis is rarely made. Ultrasound, computed tomography, endoscopy, capsule endoscopy, intraoperative endoscopy, deep enteroscopy (with single or double-balloon enteroscopy or spirusenteroscopy), laparoscopy, radio-tagged erythrocyte bleeding scans, and selective mesenteric arteriography have been used with increasing success for detection. Many lesions, however, can go undetected. The diverticula can quickly empty and become invisible if the diverticular ostium is large. Small diverticula may not hold contrast medium as well as larger diverticula, or they may not be filled at all. For certain diagnosis of asymptomatic and complex diverticula, laparotomy remains the gold standard.

Pneumoperitoneum is a common complication of diverticula perforation. Pneumoperitoneum without perforation or indications of peritonitis, on the other hand, is uncommon. The outcome of a rapidly closed leaking diverticulum or the transmural passage of air via a thin-walled diverticulum has been postulated as the cause of this spontaneous, generally asymptomatic pneumoperitoneum.

Nonspecific therapies such as a high-protein, low-residue diet, vitamin supplementation, antispasmodics, antidiarrheal drugs, antacids, and analgesics are used in the conservative treatment of uncomplicated patients [7,12], with success rates ranging from 46 % to 75 % [7]. The majority of investigators who discovered ileal diverticula during laparotomy did not perform resection [12]. However, some authors advocated for surgical intervention, claiming that resections performed on patients with chronic pain or malabsorption yielded positive effects [5]. Multiple jejunal diverticula have prompted resection of up to 75 cm of the small bowel, which has provided symptom alleviation; nevertheless, recurrence of symptoms is more common in these individuals (53 % vs. 17 %) [7]. For perforated jejunal diverticular disease, hemorrhage, or abscess formation following failure of a short course of bowel rest and antibiotics, resection of the afflicted area with primary jejunojejunal anastomosis is the surgical option of choice. To avoid short bowel syndrome, the surgeon should avoid major intestinal resections if at all possible.

Pneumatosis intestinalis (PI) is an uncommon but important condition in which gas is found in a linear or cystic form in the submucosa or subserosa of the bowel wall and rarely muscularis layer is involved. It is common in jejunum followed by ileocecal region and colon. Overall incidence is 0.03 % based on autopsy series [13]. PI has been found in several distinctive clinical settings: 1) in premature infants with necrotizing enterocolitis; 2) in adults with obstructive pulmonary disease; 3) in adults and children with a wide variety of associated conditions, including pyloric stenosis, jejunoileal bypass, progressive systemic sclerosis, transplantation, ischemic bowel, and drug therapy, particularly steroids, chemotherapy, and immunosuppression; 4) in adults as a primary benign problem; and 5) as an incidental finding in endoscopic mucosal biopsies.13) Pneumatosis cystoids intestinalis can be found incidentally in asymptomatic patients, while some cases presented as abdominal pain, diarrhea, abdominal distention, constipation, bloody stool, flatus, loss of appetite, weight loss, and even life-threatening illnesses including bowel necrosis and perforation. The occurrence of pneumatosis cystoides intestinalis or subserosal dissection of air around the colon or adjacent structures has been linked to pneumoperitoneum. Several reports of endoscopic ultrasonography (EUS) in the evaluation of bumps in the colon have clarified the diagnosis of PCI, while computed tomography scan is regarded as the most sensitive imaging modality for detection [14]. However, some patients who frequently presented with non-specific gastrointestinal symptoms were often prone to be misdiagnosed or maltreated and even underwent surgical resection, which resulted in several adverse events. Once life-threatening illnesses such as bowel necrosis, perforation, and infections are excluded, patients symptomatic from the cysts may be treated with oxygen and or antibiotics.

On literature review, there are case reported of both diverticular disease and pneumatosis intestinalis giving rise to pneumoperitoneum separately as well as both present in same patient involving same segment of bowel however in our case small bowel diverticulum and pneumatosis intestinalis involved different segment of bowel.

## 4 Conclusion

Finally, although jejunal diverticula with pneumatosis are uncommon, they should not be ignored. Jejunal diverticula and pneumatosis intestinalis both are rare cause of pneumoperitoneum. Combination of both the condition giving rise to pneumoperitoneum is extremely rare. These conditions can give rise to diagnostic dilemma in clinical practice. Conservative management is the first line of treatment for patients with uncomplicated symptoms such as chronic abdominal discomfort or malabsorption due to jejuno-ileal diverticulosis. Surgical resection, on the other hand, is the preferable option when the patient does not respond to conservative treatment or when complications arise. One should always think these conditions as differentials when patient with pneumoperitoneum are encountered.

## Ethical approval

Ethical approval is exempt/waived at our institution (Tribhuwan university teaching hospital).

## Funding

None.

## Author contribution

Milan KC, Suman Dahal, Laligen Awale, Prasan B S Kansakar: Study concept, Data collection, and surgical therapy for patient.

Milan KC, Suman Dahal, Sangam Shah: Writing and original draft preparation.

Milan KC, Suman Dahal: Editing and writing.

Prasan B S Kansakar, Laligen Awale: senior author and manuscript reviewer.

## Research registration number

Not applicable.

## Consent

Written informed consent was obtained from the patient for publication of this case report and accompanying images. A copy of the written consent is available for review by the Editor-in-Chief of this journal on request.

## Provenance and peer review

Not commissioned, externally peer reviewed.

## Declaration of competing interest

No conflict of interest.

## Data Availability

All the required information is in manuscript itself.
