# Supplementary figures and images for: Jejunal diverticulum and pneumatosis intestinalis presenting as pneumoperitoneum: A case report
Source: Int J Surg Case Rep. 2023 May 13;107:108320. doi: 10.1016/j.ijscr.2023.108320 (PMC10205454; doi:10.1016/j.ijscr.2023.108320)

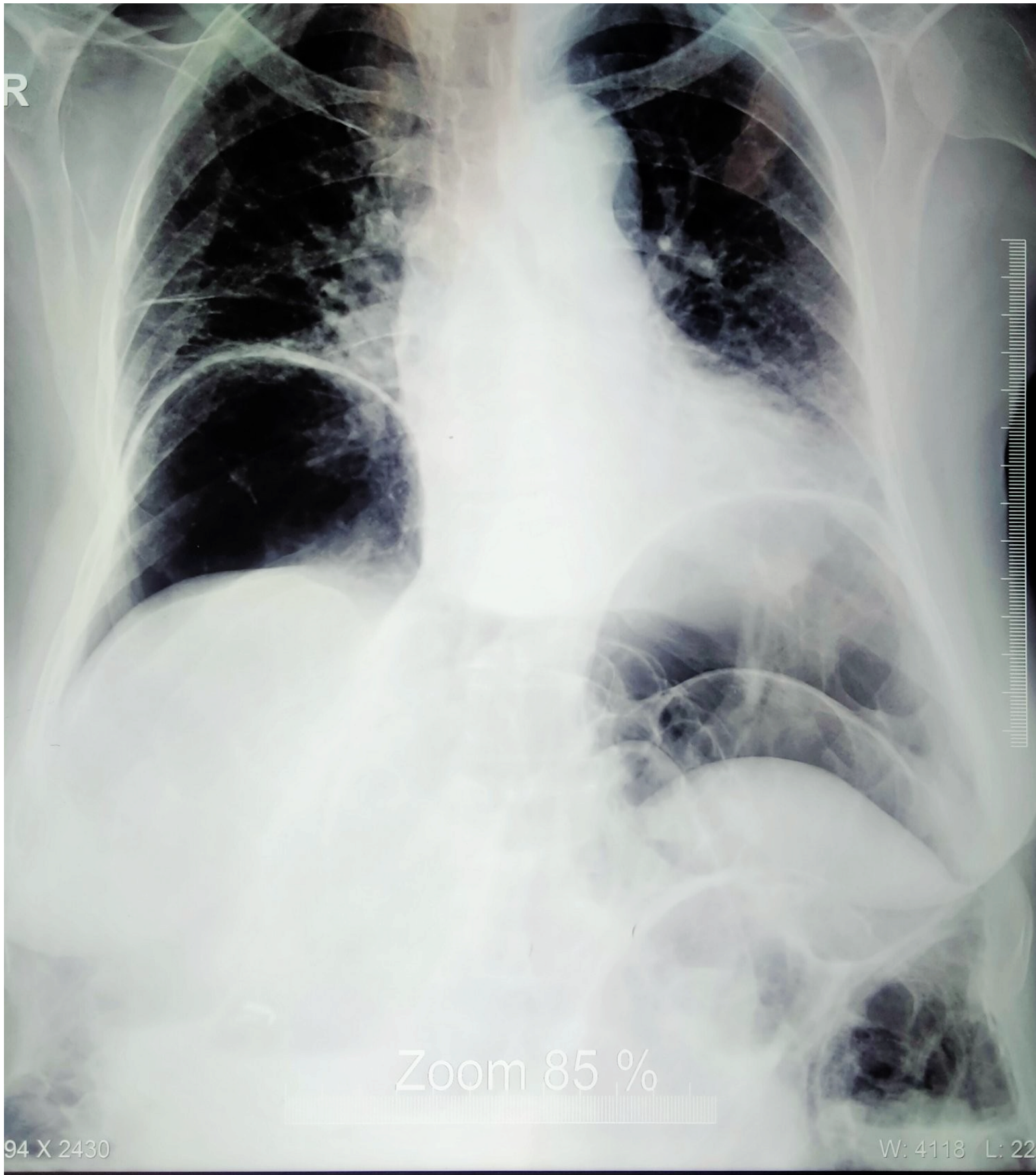

Supplement: Supplementary Fig. 1 [file mmc1.pdf]

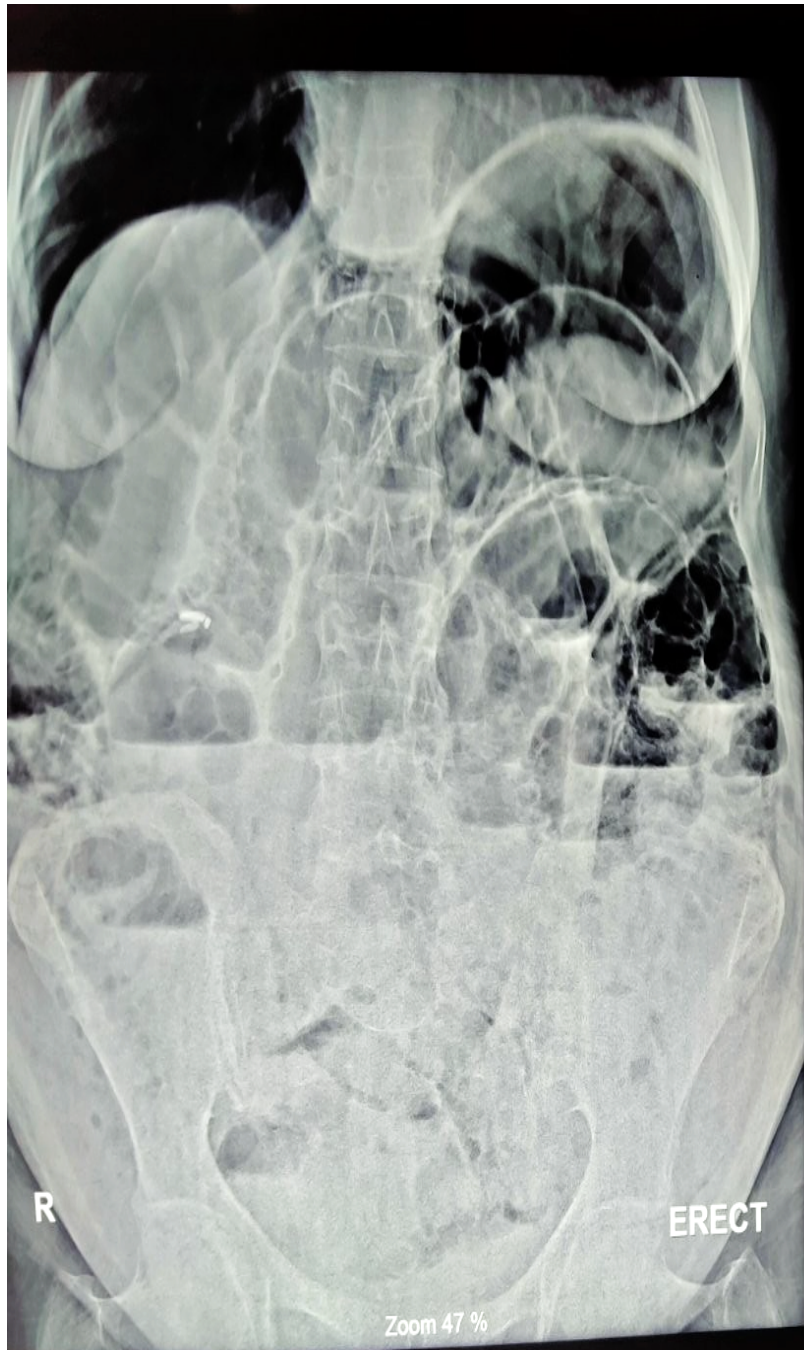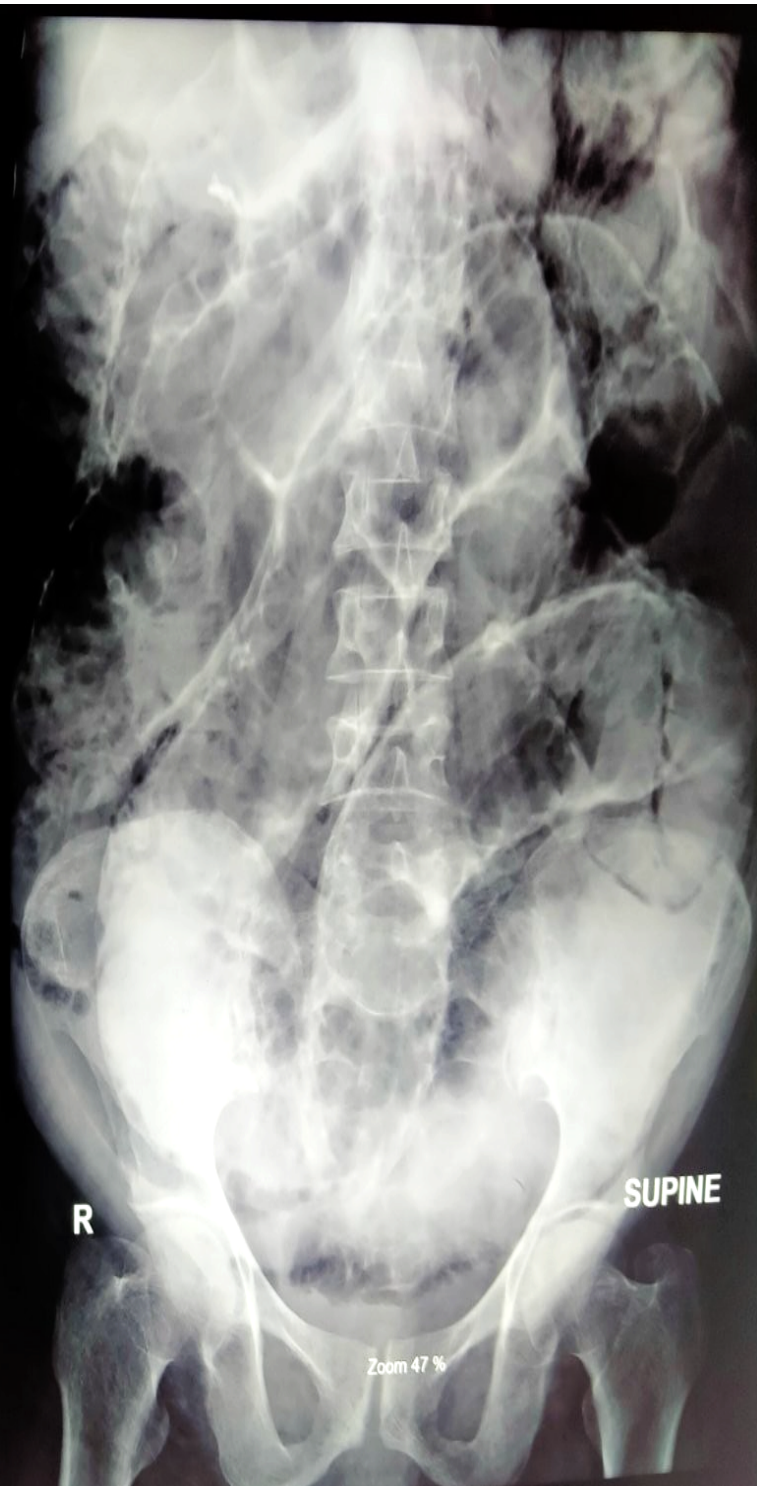

Supplement: Supplementary Fig. 2 [file mmc2.pdf]

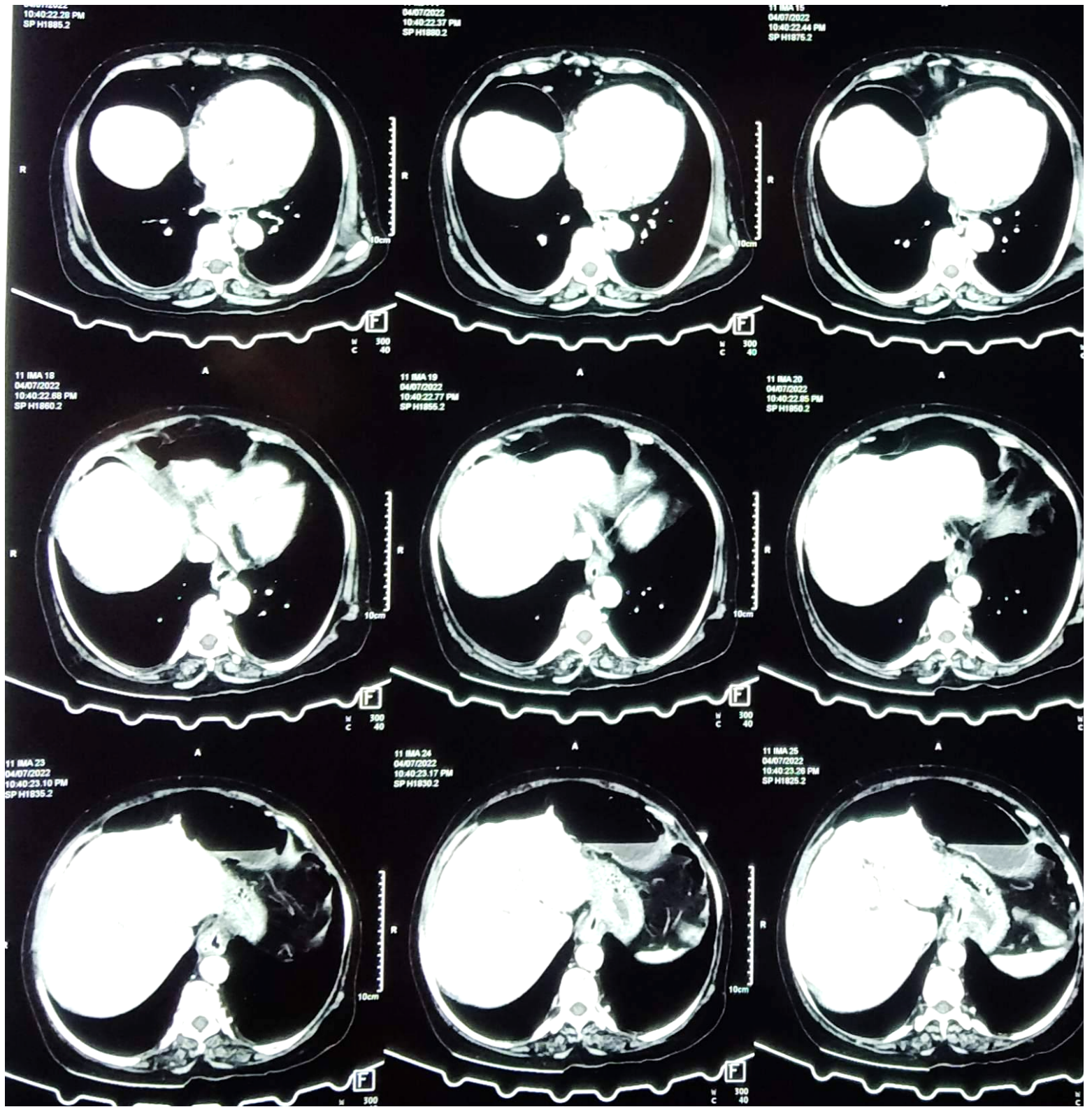

Supplement: Supplementary Fig. 3 [file mmc3.pdf]

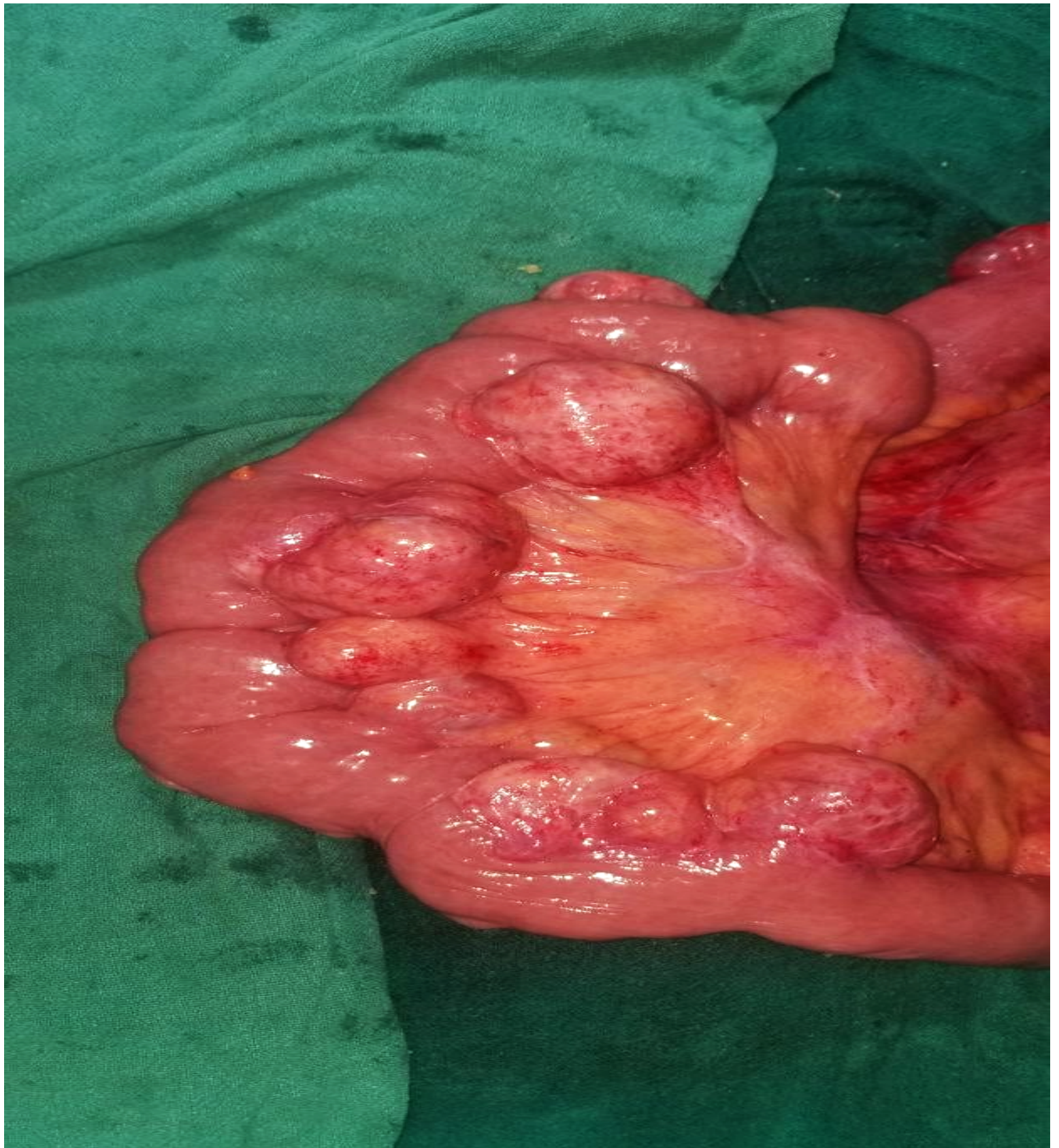

Supplement: Supplementary Fig. 4 [file mmc4.pdf]

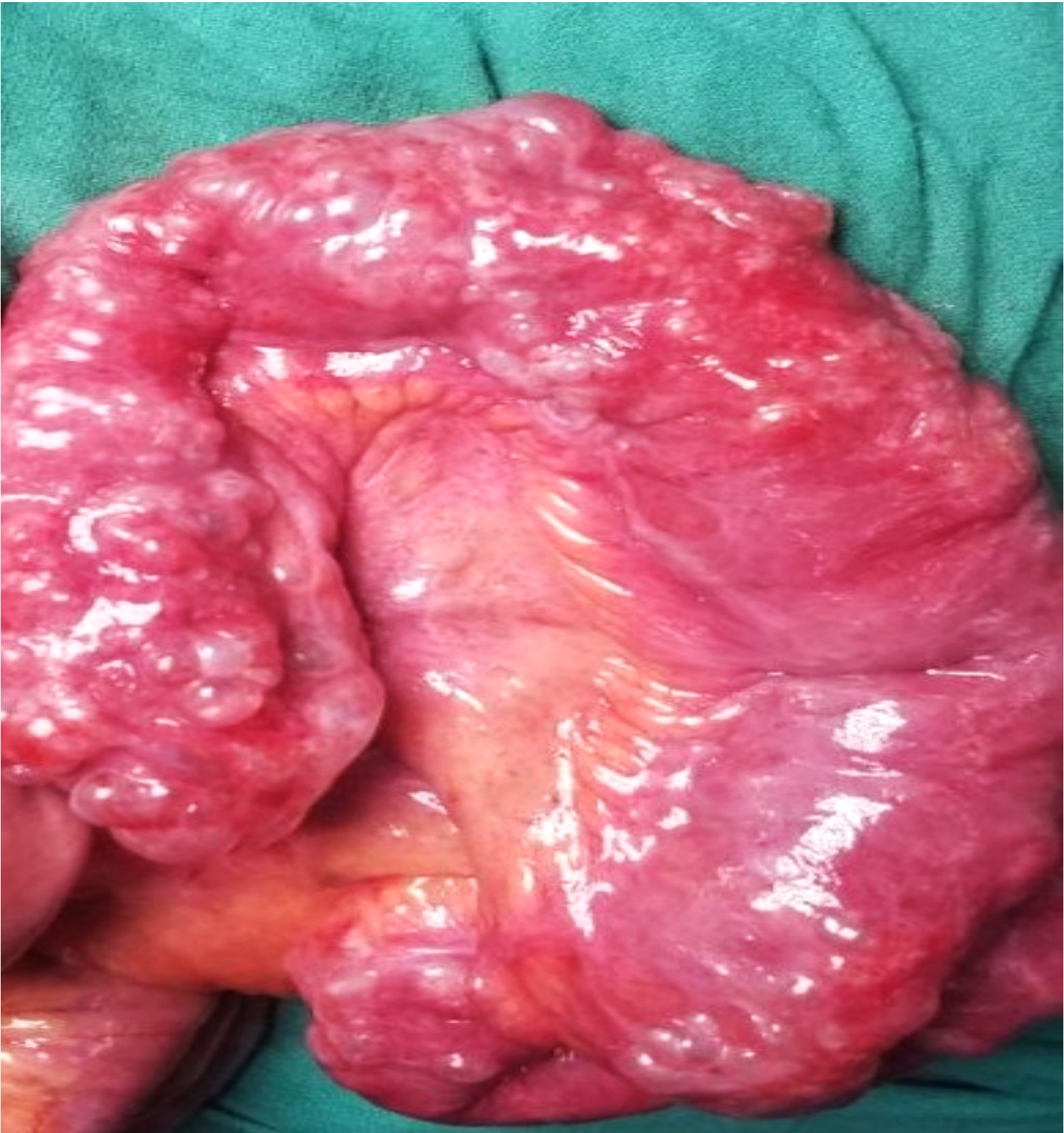

Supplement: Supplementary Fig. 5 [file mmc5.pdf]

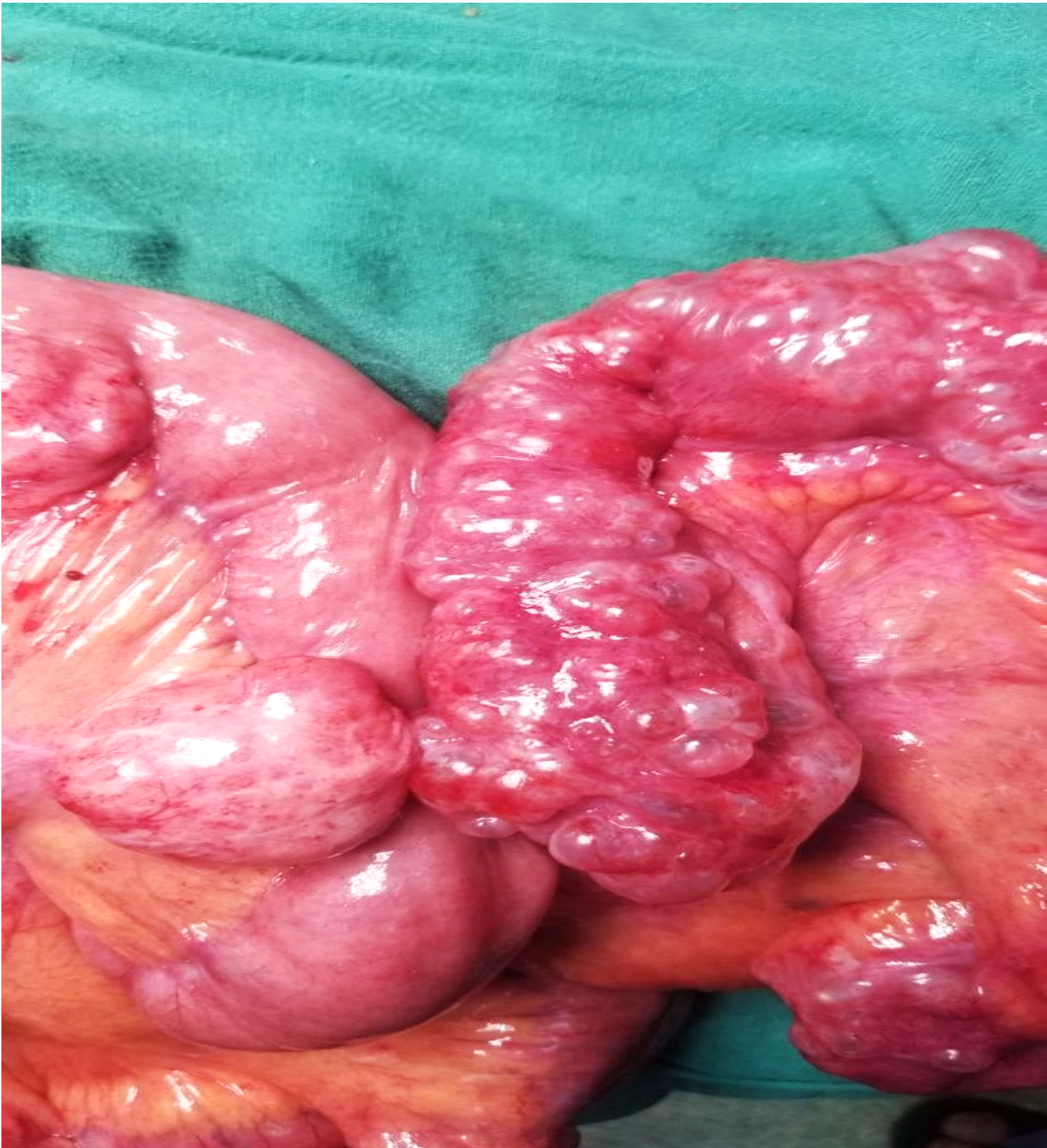

Supplement: Supplementary Fig. 6 [file mmc6.pdf]

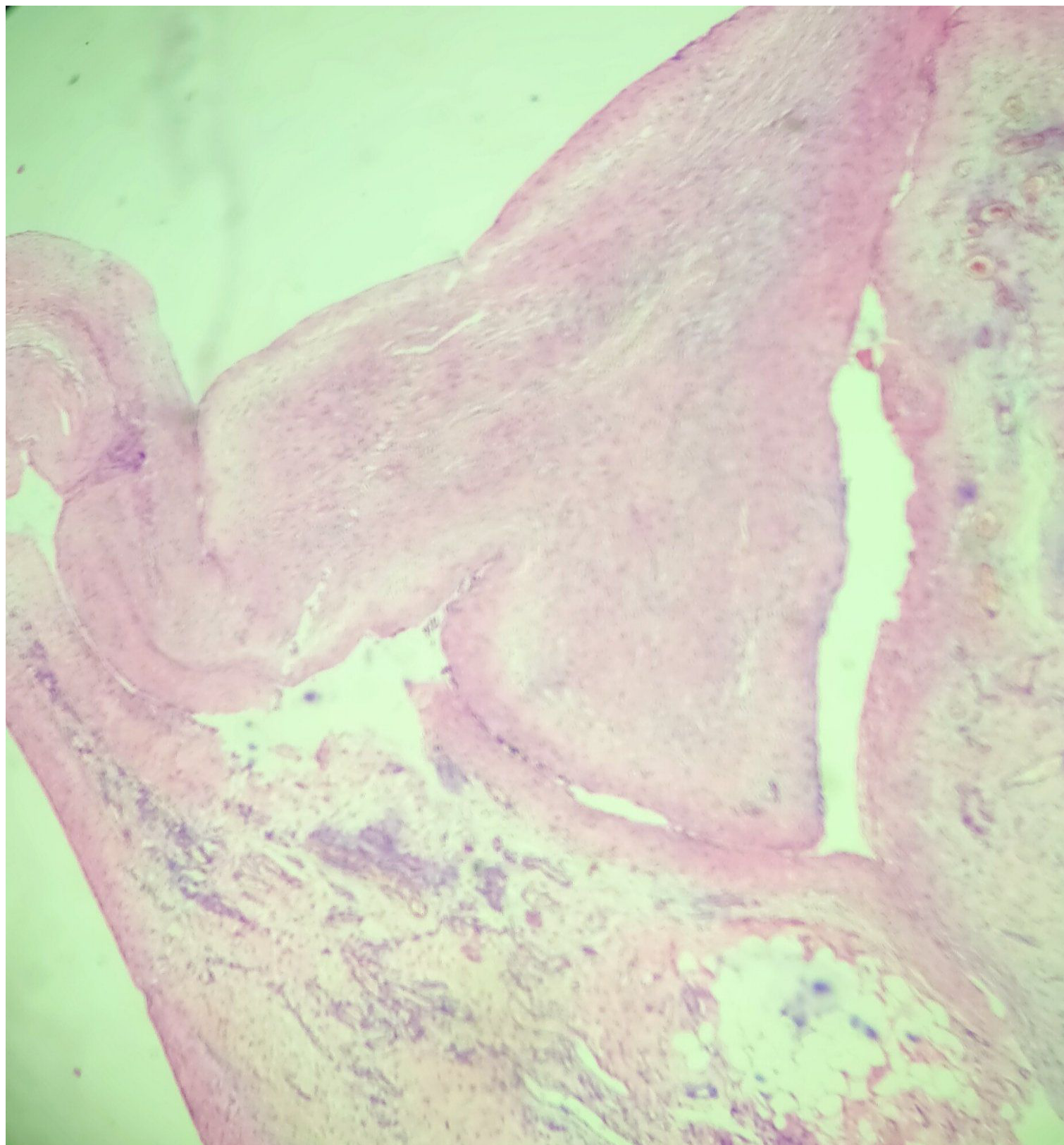

Supplement: Supplementary Fig. 7 [file mmc7.pdf]

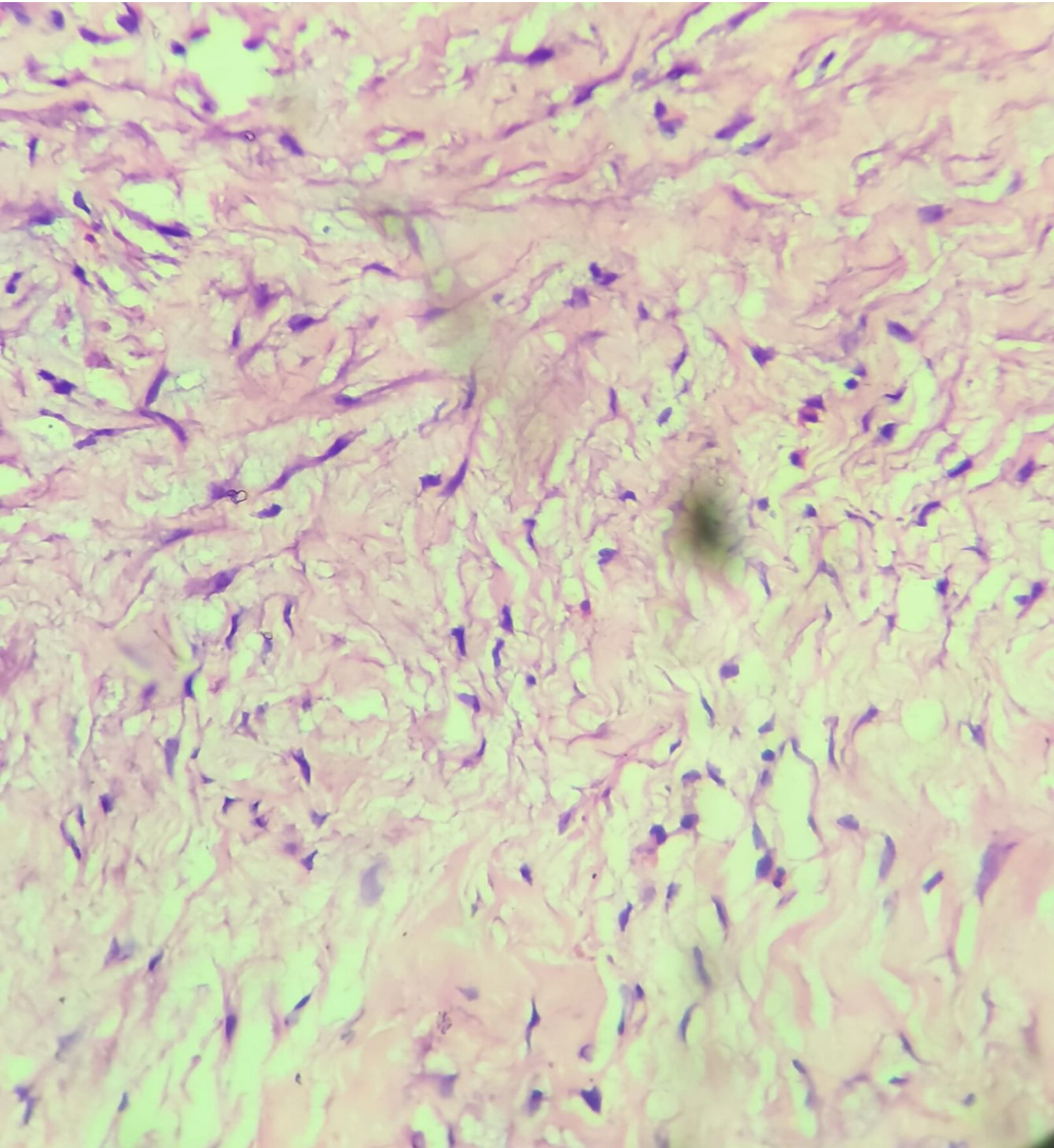

Supplement: Supplementary Fig. 8 [file mmc8.pdf]
